# Experiences of Informal Caregivers Facing Cancer While Caring for Someone With Dementia: Qualitative Interview Study

**DOI:** 10.2196/93688

**Published:** 2026-10-05

**Authors:** Elizabeth Travis, Mollie Louise Price, Millie Slessor, Jacqueline Crewe, Laura Ashley

**Affiliations:** 1 Leeds Institute of Health Sciences, University of Leeds, Worsley Building, Leeds, England, LS2 9JT, United Kingdom, 0113 343 0839; 2 School of Humanities and Social Sciences, Leeds Beckett University, Leeds, England, United Kingdom; 3 University of Staffordshire, Stoke-on-Trent, England, United Kingdom

**Keywords:** caregivers, older people, dementia, cancer, multimorbidity, qualitative research

## Abstract

**Background:**

Informal caregivers play a fundamental role in the diagnosis, treatment, and care received by their family members. Caring for someone with dementia is particularly burdensome and can have negative effects on caregivers’ health. Despite caregivers being widely recognized as vulnerable, the psychosocial needs of caregivers who are managing cancer themselves remain unknown.

**Objective:**

This study aims to investigate the experiences of UK family caregivers who have received a cancer diagnosis while caring for someone with dementia or memory problems.

**Methods:**

Twenty-five UK informal family caregivers who had received a cancer diagnosis were recruited through convenience sampling to take part in in-depth semistructured interviews. The interviews explored participants’ experiences of managing cancer alongside dementia caregiving, including the impact on treatment and caregiving, emotional challenges, support needs, and experiences of formal and informal support. Ethical approval was obtained from the Leeds Beckett University Research Ethics Committee, and all participants provided informed consent. Reflexive thematic analysis was conducted.

**Results:**

Three key themes were identified: (1) caring responsibilities can complicate and take priority over cancer care, (2) caregiving can intensify cancer-related distress and anxieties, and (3) managing cancer while caring increases the need for support and coping resources. For dementia caregivers, having cancer can surface/heighten feelings of predeath grief and worries about future care planning for their care recipient. Dementia caregivers viewed multifaceted social support as vital for enabling access to and coping with cancer treatment.

**Conclusions:**

Dementia caregivers who are also living with cancer continue to shoulder the burden of illness work on behalf of their care recipients while simultaneously managing their own cancer diagnosis and treatment. Caregiving responsibilities often take priority over cancer care, intensify the practical and emotional challenges of cancer, and increase the need for and reliance on support resources. These findings highlight the need for person-centered adjustments to cancer care, dementia-friendly health care environments, and tailored psycho-oncology support that address the practical and emotional needs of dementia caregivers.

## Introduction

An informal caregiver is a person who provides unpaid care to a family member or friend with a health condition or illness [1]. In the United Kingdom, 5.8 million people identify as informal caregivers [2], and according to Carers UK, the number of unpaid caregivers in the United Kingdom is much higher than reported because many people do not recognize themselves as a caregiver [3]. Informal caregivers play a fundamental role in the diagnosis, treatment, and care received by their family members. Many informal caregivers provide care for individuals living with multiple long-term health conditions, which can increase caregiving demands and burden [4,5]. They provide physical, emotional, social, and financial support on a daily basis to care recipients and are often responsible for aiding communication of care recipients’ medical needs with health care professionals [4,6]. Recent research has highlighted the support needs of caregivers of people with cancer and dementia, helping to inform and improve interventions and services provided to them [4]. A scoping review of 34 studies highlighted common practical difficulties faced by caregivers of someone with multimorbidity, including having to manage multiple health care teams, appointments, and medications [7]. Notably, caregivers of people with dementia have reported practical difficulties with attending medical appointments due to “dementia-unfriendly” hospital settings [8].

Informal caregivers are 16% more likely than noncaregivers to live with 2 or more long-term health conditions [1], and there is a growing amount of evidence that unpaid care should be considered a social determinant of health [1]. Caring for someone with dementia is particularly burdensome (eg, it can involve constant supervision, emotional stress, and physical exhaustion), leading to known negative effects on caregivers’ health [9-11]. Despite caregivers being widely recognized as vulnerable, those caregivers who are managing cancer themselves remain an underresearched group [12]. To our knowledge, no research has been conducted to investigate the experiences, difficulties, and support needs of informal caregivers who receive a cancer diagnosis and treatment themselves. This qualitative study therefore aimed to investigate the experiences of UK family caregivers who have received a cancer diagnosis while caring for someone with dementia or memory problems. The study aimed to specifically identify the difficulties and issues faced by patients with cancer and existing gaps in psychosocial support. Any cited recommendations for interventions, policy, and practice will also be reported and considered.

## Methods

### Participants

Participants were people with experience of simultaneously receiving a cancer diagnosis and caring for someone with dementia. Current or former caregivers were eligible if they had, within the last 5 years, provided unpaid informal care to a family member living with dementia or memory problems; had during this time also been diagnosed with cancer; were aged 18 years or older; were living in the United Kingdom; and were English literate. People who had a relative with dementia who had recently passed away (in the last 3 mo) were advised not to take part. None of the participants dropped out or withdrew from the study. The reporting of the current study adheres to the COREQ (Consolidated Criteria for Reporting Qualitative) guidelines [13].

### Sampling and Recruitment

Participants were recruited through convenience sampling using community-based advertisements via relevant charities and support groups across the United Kingdom and the Join Dementia Research platform hosted by the National Institute for Health and Care Research (NIHR). Interested caregivers contacted the research team for more information. For those who wished to proceed, a discussion via email or telephone took place to allow the researcher to check the participant’s eligibility.

### The Research Team

The research team comprised four nonclinical women researchers and a women dementia support advisor/research coordinator working for a local dementia charity. The team collectively had considerable experience and expertise in applied psychosocial health research, including undertaking qualitative interview studies with informal caregivers and with people affected by cancer, dementia, and the cooccurrence of these conditions. The study was prompted by concerns raised by the dementia charity and National Health Service (NHS) oncology staff, in informal conversations with a member of the research team, regarding the challenges faced by dementia caregivers undergoing cancer treatment. Relevant personal and professional experiences within the research team included family experiences of dementia and cancer, alongside research expertise in dementia support and behavioral oncology. Consistent with reflexive thematic analysis (RTA), researchers reflected on how their experiences and perspectives may influence data interpretation through regular team discussions during coding and theme development.

### Data Collection

Participants took part in one individual, audio-recorded, semistructured interview undertaken by ET via telephone or video call, depending on participant preference. Interviews explored participants’ experiences of caring for someone with dementia alongside being diagnosed with cancer themselves. The interview topic guide (Multimedia Appendix 1) was informed by previous research in this area, including by our team (LA and MLP). The interview topic guide was reviewed and edited by the research team. The first interview was treated as a pilot, and no substantive changes to the topic guide were required. Interviews were conducted flexibly, allowing participants to discuss issues that were most important to them. Sensitive topics, such as future care planning, end-of-life concerns, and experiences of grief and loss, were raised by participants themselves rather than through direct questioning. All interviews were conducted by ET, an experienced qualitative researcher with expertise in psychosocial applied health research. The interviewer encouraged participants to discuss experiences that were important to them, and if participants became upset, they were reminded that they could pause, take a break, or continue the interview at a later time. The interview approach remained consistent throughout the study. Participants first provided sociodemographic information (eg, gender and age) and shared their experiences of caring for someone with dementia and how they came to find out they had cancer. Participants were recruited from 7 of the 9 regions of England and 1 region of Scotland, providing broad geographical representation within the sample. Interviews then focused in-depth on caregivers’ experiences, difficulties, and needs around simultaneously caring and facing cancer and on experienced or potential sources of support or ways of helping to address caregivers’ challenges and needs at this time.

### Data Analysis

Data were analysed using RTA [14,15]. Analysis was an inductive, iterative, collaborative process involving both independent review and team discussion of the data, with decisions made collaboratively by consensus. Analysis was begun concurrently with data collection, allowing analytical insights to inform subsequent interviews and enabling the research team to assess whether the data captured a sufficiently broad and rich range of experiences to address the study aims. Recruitment ended when the team agreed that the dataset provided adequate depth and diversity of perspectives relevant to the research aim. We adopted an experiential approach to RTA, informed by a hermeneutic phenomenological approach, as we aimed to understand how participants experienced and made sense of receiving a cancer diagnosis while caring for someone with dementia, including their support needs [16]. This approach complemented RTA by focusing on participants’ perspectives and meanings, while recognizing that theme development involved interpretation by the research team. Transcripts were analyzed following the six stages of RTA as follows: (1) initial interview observations were recorded in MS Word by the interviewer following each interview. Interviews were then transcribed and anonymized by ET (P1-P19) and MS (P20-P25). (2) Codes were generated inductively and independently by ET, MLP, and JC to develop an analytical coding framework, which was subsequently applied to all transcripts by ET. In developing codes, MLP and JC each coded a different sample of 6 transcripts, which were also all coded by ET. (3) Similar codes were grouped and candidate themes and subthemes generated by ET. (4) ET, MLP, and MS met to review and refine candidate themes and subthemes and select indicative quotes. (5) ET and LA met to further review, restructure, and define themes and subthemes and determine the illustrating quotes. (6) Final theme and subtheme titles, descriptions, and illustrative quotes were reviewed and agreed upon by team consensus (MLP, MS, and LA). All stages of coding and data analysis were carried out in MS Excel. Participant validation (also known as member checking) was not undertaken in this study. Instead, the credibility of the analysis was supported through iterative discussion and interpretation of the data within the research team.

### Ethical Considerations

The study received ethical approval from the Leeds Beckett University Psychology Local Research Ethics Committee on March 14, 2024. All participants received written information about the study and provided informed consent prior to the interview.

## Results

### Participants and Data Collection

Twenty-five informal family caregivers participated in the study. Sample characteristics are summarized in Table 1. No participants withdrew from the study. Interviews were conducted by telephone (n=15) or online video call (n=10) between March 25, 2024, and January 15, 2025, and ranged from 24 to 78 (mean 47.72, SD 12.92) minutes in duration. Variation in interview duration is likely a reflection of individual differences in participants’ experiences, the amount of detail they wished to share, and their characteristic verbal style; however, factors contributing to interview length were not assessed.

**Table 1.** Sample characteristics.

Variables	Count (N=25)
Age (y), mean (SD; range)	68.32 (10.95; 46-81)
Gender, n (%)	
Women	14 (56)
Men	11 (44)
Ethnicity, n (%)	
British–White	23 (92)
British–South Asian	1 (4)
Norwegian–White	1 (4)
Relationship to care recipient, n (%)	
Spouse/partner	12 (48)
Adult child	12 (48)
Child-in-law	1 (4)
Socioeconomic status: index of multiple deprivation	
Quintiles	
1	2 (8)
2	1 (4)
3	4 (16)
4	6 (24)
5	7 (28)
Unknown (post code not specified)	5 (20)
Residing with care recipient, n (%)	
Yes	12 (48)
No	13 (52)
Current employment, n (%)	
Employed	5 (20)
Unemployed	2 (8)
Retired	18 (72)
Care recipient dementia diagnosis, n (%)	
Vascular dementia	2 (8)
Alzheimer disease	14 (56)
Frontotemporal dementia	3 (12)
Mixed dementia	3 (12)
Parkinson disease dementia	1 (4)
Memory problems (awaiting formal diagnosis)	1 (4)
Not specified	1 (4)
Caregiver cancer diagnosis, n (%)	
Bowel	2 (8)
Bladder	1 (4)
Breast	11 (44)
Skin	1 (4)
Kidney	1 (4)
Prostate	5 (20)
Thrombocythemia	1 (4)
Leukemia	2 (8)
Multiple myeloma	1 (4)

### Key Findings

Three themes, each with interrelated subthemes, were developed from the data to capture the experiences of and support received by informal caregivers while having cancer. The findings highlight how caregiver identity and role shaped and intensified both the practical and emotional challenges of cancer, giving rise to multifaceted support needs. Caring responsibilities were central to how participants experienced cancer, often taking precedence over their own health needs and influencing engagement with their treatment. Competing caregiving demands produced both practical disruptions to engaging in cancer care and intensified emotional consequences of facing cancer, including grief and loss, and anxieties about future care for the person living with dementia. In response, participants described a need to draw significantly on practical, financial, and psychological resources to effectively manage the demands of caregiving and cancer.

### Theme 1: Caring Responsibilities Can Complicate and Take Priority Over Cancer Care

#### Overview

Caregivers spoke about their cancer as a competing priority to their caring responsibilities. Caring responsibilities frequently acted as the priority around which cancer was managed, shaping engagement with treatment and health care services. Interviews highlighted how caregiver burden and related responsibilities can complicate and negatively impact care accessibility and experiences across the cancer continuum.

#### Competing Caring Demands Make Cancer Care More Difficult to Manage

Caregivers cited various difficulties experienced in managing the demands of their dual role as a caregiver and people with cancer. These complications made undergoing cancer treatment difficult and stressful for some caregivers. Logistical complications included the need for caregivers to coordinate attendance at multiple appointments and treatments, the reality of having little to no choice but to prioritize the person with dementia over their own treatment, and the challenges arising when care recipients walked away from the waiting area during their own appointments or treatment.

For many caregivers, managing their cancer treatment required considerable planning and coordination alongside upholding existing caregiving responsibilities.

I’ve moved appointments and blood tests, or we’ve done it all on the same day. And you know, the other week we visited three hospitals in the region of England, two for my mum and one for me.

[P7, age:56, daughtercaregiver]

Challenges were not limited to scheduling appointments and could also create concerns about the safety and supervision of the person living with dementia.

*She sat in the waiting room. Unfortunately, it was an hour long before I could come out. When I came out, she wasn’t there. So, I said to the receptionist “my wife?,” “Oh yes,” she said. “She went to the toilet some time ago and she hasn’t come back,” so I was up and down the hospital searching for her, so I never took her again.*

[P4, age:79, husband-caregiver]

#### Prioritizing Caring Responsibilities Can Affect Engagement With Cancer Care

From cancer diagnosis to outcome, the role of being a caregiver continued to be at the forefront of caregivers’ minds, viewed as the greatest need before their own cancer. Examples cited demonstrate how being a caregiver with cancer can have a detrimental impact upon the patient’s cancer trajectory in terms of help-seeking, decision-making, ability to give their full attention when attending medical appointments, and the need to reset and recover from their own treatment. As expected, the caregiver’s burden and challenges were thought to be greater when the care recipient’s dementia was at an advanced stage rather than when it was at an earlier stage.

For some participants, caregiving responsibilities took priority over their own health needs and delayed help-seeking for cancer symptoms.

*I would say that I put off going about the lump when it came back last year…my dad was in hospital at the time, and so we were up and down and his circumstances of him going into hospital.*

[P10, age:61, daughter-caregiver]

*They were coming to appointments with me, and I found that I was more concerned about them and their reactions than I was about myself. I couldn’t listen. I was looking at their faces and you know, they had all sorts of questions.*

[P2, age:73, wife-caregiver]

Caregiving responsibilities also influenced how some participants approached their treatment decisions, with a preference for options that would minimize disruption to their caregiving role.

*Radiotherapy offered a chance that it might not knock me out so much and all the rest of it, compared to the recovery time from an operation, so I could carry on caring.*

[P6, age:80, husband-caregiver]

### Theme 2: Caregiving Can Intensify Cancer-Related Distress and Anxieties

#### Overview

Beyond its practical impact on cancer care, being a dementia caregiver also shaped and intensified the emotional sequelae of cancer for caregivers. Caring for a person with dementia while facing cancer often intensified caregivers’ feelings of loss, uncertainty, and vulnerability, leading to our second theme: caregiving can intensify cancer-related distress and anxieties. Being a caregiver for someone with dementia often meant that caregivers experienced additional or heightened cancer-related distress and anxieties tied to the losses and responsibilities of their caregiving role. Caregivers’ realization of this emotional distress often occurred and was only dealt with at a later stage in their cancer journey. Caregivers with a terminal diagnosis worried over practical arrangements and the emotional burden of who would assume the challenging dementia care responsibilities after their death.

#### Cancer Triggers Feelings of Loss and Grief Toward the Person With Dementia

Being diagnosed with cancer and undergoing treatment surfaced feelings of loss in caregivers. This was attributed to their predeath grief toward the care recipient, who, due to their dementia, could no longer provide them with the emotional support they needed. Caregivers explained that they were unable to turn to their care recipient in the way they once could, before the care recipient’s dementia, for emotional support. For some caregivers, this unmet need for emotional support to help them deal with their cancer and feelings of predeath grief was only realized retrospectively and acted upon at a later stage or following cancer treatment.

Adult-child caregivers in particular described a sense of loss arising from no longer being able to rely on their parent, the care recipient, for emotional support in the way they had before dementia.

*I would have probably liked to talk about it to my mum and dad. You always go to your mum don’t you. I missed that.*

[P14, age:66, daughter-caregiver]

*I said “Dad, I’ve just got a diagnosis of cancer”, and his reaction to that, imagine this...your son tells you, “I’ve got cancer, it’s not a little cancer, it’s a serious cancer”. And there was a pause. And he said, “oh, I’m sorry to hear that. Is there anything we can do?” that was literally what he said. It was so unemotional and uncaring.*

[P9, age:59, son-caregiver]

For some, the emotional impact of having cancer was only recognized later, once the immediate demands of caregiving and their cancer treatment had passed.

*I don’t think I actually acknowledged...making the decision to not tell my mum was one thing, which was upsetting, but then I had to sort of put that in the box because I’ve got this to deal with now [having cancer]. It’s only really in the last year or so that’s sort of gone, do you know that was really actually quite bad. And yeah, you know what I’ve missed out on and how I actually really needed my mum. But at the time I had to put that into a box because I can’t deal with that now because I’d got this [having cancer] to deal with. What was happening with mum was one thing and what was happening with me, was another thing. And I didn’t connect them at the time, because it was too upsetting to connect them probably. So I never spoke to anybody about it because it’s not something I was thinking about.*

[P18, age:49, daughter-caregiver]

#### Caring Heightens Cancer-Related Anxieties and Fears About Dying and Future Planning

For those caregivers with an incurable cancer diagnosis, uncertainty and a lack of control regarding timings of their own and their care recipient’s future situation were a cause of great distress. Caregivers were worried about what would happen to the person they cared for when they died. Their concerns encompassed both practical matters, such as funeral arrangements, and emotional anxieties, particularly around who would take on the responsibility for caregiving, a role that is often exhausting and burdensome when supporting someone with dementia.

*I fear for the future. I fear for what’s going to happen. In terms of caring for me or caring for <Person living with dementia name> because he’s not going to want to go into a home. He’s got quite an awkward streak in him, always has had. I think that’s probably what I fear, and for my own health I’d, you know, [Crying] I don’t know what the future holds. I’ve felt very ill before now and a year ago and you know, if I was like that, I wouldn’t be able to look after him because it is very tiring.*

[P1, age:76, wife-caregiver]

*It’s terrible when you’re wishing your mum to die before you do. Because I don’t want to cause her the trauma of, because I’ve got a metastatic diagnosis, you know, gosh I’m lucky to be here now. And that is a really terrible thing, that is one of the worries.*

[P7, age:56, daughter-caregiver]

*My mom and now my sister, who’s going to look after them, if I’m not there, that’s more the fear now, because I know that my kids will be okay, and they’ll take care of their kids. But Alzheimer’s is dreadful, and we’ve had dreadful experiences with that.*

[P16, age:69, daughter-caregiver]

Managing the demanding roles of dementia caregiver and patient with cancer in tandem, along with the resultant practical and emotional challenges, was often cited as overwhelming. Consequently, participants reported a need for greater practical, emotional, and financial support, which forms our third theme.

### Theme 3: Managing Cancer While Caring Increases the Need for Support and Coping Resources

#### Overview

Many caregivers reported a lack of awareness of available dementia support services, limited availability of those services, and inconsistent health and social care for the person with dementia. To overcome systemic issues, caregivers often cited instrumental, financial, and emotional avenues of support they had depended upon to enable them to continue to care for their care recipient while also attending medical appointments and treatment of their cancer.

#### Reliance on Practical and Emotional Support From Family and Friends

Reliance on support networks was frequently cited as a response to the competing demands described in theme 1 and the emotional burden described in theme 2. Family members and friends often provided practical care that freed up participants to attend their cancer appointments, while also offering reassurance and emotional support for their cancer-related worries. All caregivers cited their reliance and sole dependence on friends and family to be able to attend medical appointments and treatment related to their cancer, particularly when caregivers had received last-minute appointments and were unable to arrange professional care at short notice. This instrumental support provided by family and friends also included respite care, cited by caregivers who at times needed a break to recharge physically and mentally. Caregivers also talked about the emotional support they had received from friends and family.

*if somebody else had looked after my dad, instead of us all the time. And given us a break, because then none of it would have been so difficult.*

[P10, age:61, daughter-caregiver]

*It didn’t really occur to me that there might be any facilities for it. So I didn’t, you know, if you’re not aware of something being available, possibly you don’t even think to look for it. If you’re not told, that would help or not.*

[P11, age:79, wife-caregiver]

These accounts suggest that limited awareness of available support services may increase caregivers’ reliance on family and friends.

*I had a bit of a meltdown. I really couldn’t see how I was going to cope. And I said to my daughter, could you have him? And so, she had him to stay for two nights, one day and two nights, and during that time I managed, I got my brain sorted really.*

[P1, age:76, wife-caregiver]

Support from family members often extended beyond practical help and included emotional support during cancer treatment and recovery.

*It’s my wife who has taken the real hit in terms of emotionally supporting me, yet also taking on the burden, as it were, of supporting my mother*

[P25, age:67, son-caregiver]

*They [local council] didn’t say they weren’t interested, but they just kept saying, oh, we’re fully booked. I don’t know that we can do that on the day you want, and because I wasn’t getting a lot of notice to go into hospital, so I was sort of saying to them, you know, in about three or four days time. So in the end, the family just said, stop messing about with it, although it sounds a good procedure.*

[P4, age:79, husband-caregiver]

#### Financial Security Helps Caregivers Manage Cancer and Caring Responsibilities

Financial resources appeared to help mitigate the impact of challenges described in themes 1 and 2. Participants with access to savings, pensions, or privately funded care were often better placed to negotiate competing caregiving and cancer demands, reducing some of the practical tensions between caring and their cancer treatment. Given the current issues caregivers cited with a lack of health and social care provided, many caregivers felt privileged and fortunate that they had the option to fund private care themselves through their own or family personal finances. For some, personal finances had enabled them to reduce their working hours or take early retirement. For caregivers without personal finances to draw upon, this was viewed by caregivers interviewed as an unimaginable problem to cope with and manage.

*Just to make sure they’re safe and you don’t have to worry about it. Do you know what I mean? Everything that you’re going through without having to worry about everybody else. That would be the ideal world. But there’s no budgets anywhere, is there for that.*

[P13, age:53, daughter-caregiver]

*Especially for people who haven’t got the money, yeah, it must be so difficult. I just feel so completely privileged.*

[P5, age:77, wife-caregiver].

Financial security enabled some caregivers to reduce the demands of paid work, preserving more of their personal resources for managing the demands of caregiving and their cancer treatment.

*Since I had cancer last year, I have only gone back to work, I halved my shifts, so that is a considerable difference and I am really aware of that but, we haven’t, you know, we’ve managed. No, no, we’re lucky.*

[P10, age:61, daughter-caregiver]

*We’ve both got private pensions, and we got somebody round and realized that we could take a bit of that every month and not have to, to carry on working if we didn’t need to, for the short term at least.*

[P19, age:59, daughter-caregiver]

#### Personal Resilience Supports Coping With Cancer and Caregiving Demands

Caregivers’ accounts highlighted emotional resilience as key to how they actively coped with the practical and emotional challenges described in themes 1 and 2. Many caregivers spoke in ways that suggested they had a positive mindset about how they perceived their situation and challenges they faced. To help with coping, caregivers appeared to engage in benefit finding*,* somewhat indicative of their underlying resilience, which not only helped them deal with challenges but also appeared to strengthen their resilience further. For example, some caregivers viewed an early cancer diagnosis as a blessing, given that it aligned with their perception of the illness as being less severe. Being a caregiver was viewed as a positive distraction, a role that gave caregivers a sense of purpose and provided a good distraction from worrying too much about their own cancer. One caregiver explained how she tries to focus on today, practicing mindfulness and resilience as a way to overcome and cope with any worries about what tomorrow will bring.

*I think having my husband to look after was a distraction from focusing too much on the cancer and kind of getting panicky, so in a way, it just gave me something else to focus on as well. I think if it had just been me and my cancer, I would have focused more on that and then that would have been maybe harder to keep any sort of anxiety out of the way.*

[P5, age:77, wife-caregiver]

Caregivers often described actively focusing on the positive aspects of their situation as a way of coping with the challenges they faced.

*The cancer was caught at a very early stage. So, fortunately the cancer never really figured it was, just. Oh, look, I’ve got to do X, it was no more a problem than going to the dentist.*

[P3, age:76, husband-caregiver]

*Having cancer is not really an issue in the whole thing of caring for her because it’s a non-event almost.*

[P22, age 79, husband-caregiver]

Other caregivers described focusing on the present rather than worrying about the future as an important coping strategy.

*It is a progressive disease, I do know what the future holds, but, I don’t think about the future, we live for today. you know, I try to live as normal life as possible. Think about today, let tomorrow take care of itself, really. That’s the way I’ve coped really, do I have wobbles, yes I do.*

[P15, age:80, husband-caregiver]

## Discussion

### Key Findings

To our knowledge, this is the first qualitative study to explore, in the United Kingdom and internationally, the experiences of informal caregivers who have received a cancer diagnosis while caring for someone with dementia or memory problems. The study aimed to specifically identify the difficulties and issues faced by patients with cancer and existing gaps in psychosocial support. The findings corroborate and extend the work by Price et al [4,17] on the psychosocial support needs of caregivers of people with comorbid dementia and cancer, while highlighting the distinct experiences of individuals who are themselves living with cancer while also caring for a person with dementia. Study findings were developed into three key themes: (1) caring responsibilities can complicate and take priority over cancer care, (2) caregiving can intensify cancer-related distress and anxieties, and (3) managing cancer while caring increases the need for support and coping resources. Furthermore, this study is the first to present evidence that caregivers living with cancer themselves continue to shoulder the burden of illness work on behalf of their care recipients. Illness work, as described by Corbin and Strauss [18], refers to tasks such as taking medications, monitoring symptoms, attending medical appointments, and coordinating with health care providers. Price et al [4] emphasized the relentless caring regime involved in being a caregiver, and our study findings demonstrate that the caregiver role prevails, even to the detriment of the patient’s cancer trajectory. Caregivers cited examples of how their caregiving role had influenced their decisions to opt for less invasive treatments with shorter recovery times, delayed their help-seeking for cancer symptoms, required them to reschedule appointments, and left them with limited time to recover from their own treatment. These findings extend previous research on illness work by demonstrating that caregiving responsibilities often continue when caregivers themselves are managing a serious health condition such as cancer. Consistent with wider caregiving literature, caregivers commonly continue providing care despite chronic illness, pain, and other health challenges of their own, often prioritizing the needs of the care recipient over their own health and well-being [19-22]. Participants in this study similarly placed the needs of the person with dementia ahead of their own, suggesting that the caregiver role remained of greatest importance despite cancer diagnosis and treatment.

This study underscores the unique emotional challenges faced by caregivers for someone with dementia who simultaneously need support for their own cancer diagnosis and treatment. Among the adult-child caregivers in our sample, participants described their parent as having previously been their significant source of emotional support in times of need. However, due to the progression of the care recipient’s dementia, they could no longer rely on them for emotional support. There is growing interest in assessing and addressing predeath or anticipatory grief experiences among dementia caregivers, with recognition that this is an underresearched issue in which people can feel unsupported [23,24]. The loss of emotional support associated with dementia progression may be particularly challenging for caregivers who are simultaneously coping with their own cancer diagnosis and treatment. These experiences suggest that anticipatory grief may be compounded by the emotional and practical demands of cancer, highlighting a need for psychosocial support that addresses both cancer-related distress and losses associated with dementia progression. Further work is required to better understand these intersecting experiences and support needs. Findings highlight an important need to provide targeted psychosocial support to caregivers with cancer to help them emotionally cope with and process additional levels of loss experienced due to predeath grief.

Many caregivers relied on the support from family and friends, their own financial security, and their own emotional resilience to cope. Ji et al [25] found significant correlations between psychological resilience, caregiving burden, social support, and quality of life among primary caregivers of patients with cancer. Our findings suggest that access to practical, emotional, and social support may be especially important for people balancing the dual roles of being an informal caregiver and a patient with cancer.

Our findings have implications for digital health. Caregivers reported limited awareness of available services, difficulties navigating support, and challenges balancing their cancer care with caregiving responsibilities. These needs may be addressed in part through digital solutions such as integrated patient and caregiver portals, digital signposting resources, telehealth follow-up, and online psycho-oncology support. Such approaches could help improve access to information, care coordination, and emotional support while reducing the practical burdens associated with hospital visits [26,27]. Online peer support may also be valuable, particularly for caregivers with limited support from family and friends. For example, the cancer-specific section of the Alzheimer’s Society online forum, codeveloped by Ashley et al [8,28], provides opportunities for shared understanding, emotional support, and the exchange of practical advice among people navigating both cancer and dementia caregiving. Future research could explore the acceptability and effectiveness of digital interventions for informal caregivers managing both cancer themselves and dementia-related caregiving demands.

Benefit finding is a psychological concept whereby someone searches for or identifies positive outcomes or personal growth when faced with stressful life experiences [29]. To help with coping, caregivers in this study indicated involvement in benefit finding, which not only helps them deal with challenges faced but also strengthens their resilience further. A recent review of 21 articles by Chiba et al [30] highlighted indicators of benefit finding among family caregivers of individuals with mental illness, including positive personal traits, good relationships with patients, and social support. Benefit finding has also been identified among caregivers of people with advanced cancer, where caregivers reported personal growth, stronger relationships, perceived social support, and a greater sense of meaning in life [31]. While benefit finding has been reported in other caregiver populations, our findings suggest that caregiving may also provide a sense of purpose and distraction from cancer-related concerns for some individuals. Caregivers in our study described engaging in positive distraction, focusing on their role as a caregiver, as a coping strategy, shifting their attention and concern away from their own cancer. Dobignes et al [32] investigated the use of an online positive psychology intervention to support caregivers of people with Alzheimer disease. Their findings reported that caregivers of people with earlier stage Alzheimer disease benefited the most from the intervention [28]. Further work in this area is warranted to explore and test interventions that support coping strategies for caregivers more widely. Several implications arising from our findings align with recommendations previously proposed by Ashley et al [8,28] to improve cancer care for people affected by dementia. These include cancer services identifying patients who also care for someone with dementia and considering reasonable adjustments that help them balance treatment and caring responsibilities. Flexible appointments, reduced hospital visits, dementia-friendly environments, and practical support with travel or attendance could also help minimize barriers to cancer care. It is also important to recognize the emotional impact of managing cancer while providing care. Regular discussions about well-being, access to caregivers’ support services, and tailored psychological support can help address anxiety, future care concerns, and feelings of loss or grief, many of which may be reduced through appropriate adjustments and support [8,28].

Our findings highlight the challenges of being both an informal dementia caregiver and a patient with cancer at the same time. Participants described prioritizing the needs of the person with dementia over their own health and cancer treatment, coping with the emotional impact of dementia progression intensified in the context of coping with cancer, and needing and relying on different sources of support to manage both roles. These findings suggest that the experiences of informal caregivers with cancer are not fully reflected in the existing cancer or dementia caregiving literature. Our study helps to address this gap by providing new insight into the challenges and support needs of people who are simultaneously managing a cancer diagnosis while continuing to care for a person living with dementia.

### Key Strengths and Limitations

Study strengths include recruitment of a sizeable sample with diversity across several study-relevant personal characteristics (eg, gender, age, cancer type, caregiver relationship to person with dementia, and socioeconomic status), as well as in-depth, rich data collection and collaborative, iterative data analysis involving multiple members of the research team. Limitations include the recruited caregivers who were predominantly retired and White British. Although this partly reflects the higher incidence of cancer in older adults [33] and the larger proportion of White British people affected by dementia in the United Kingdom, the Alzheimer’s Society reports that just 3% of people living with dementia are from Black, Asian, and minority ethnic communities [34].

### Conclusions

Our study demonstrates that for dementia caregivers facing cancer, caregiving responsibilities can compete with, complicate, and negatively impact engagement in cancer treatment and care. Our findings underscore the unique emotional challenges faced by caregivers for someone with dementia, who simultaneously need support for their own cancer diagnosis and treatment. For dementia caregivers, having cancer can surface/heighten feelings of predeath grief and worries about future care planning for their care recipient. Dementia caregivers viewed multifaceted social support as vital for enabling access to and coping with cancer treatment. This investigation provides initial evidence that caregivers living with cancer themselves continue to shoulder the burden of illness work on behalf of their care recipients. For dementia caregivers, accessibility and experiences of cancer care may be optimized by person-centered adjustments to care planning and scheduling and dementia-friendly hospital environments. Dementia caregivers may benefit from tailored psycho-oncology support encompassing caregiver-role concerns and needs (eg, predeath grief for their care recipient). Digital health interventions may also help address needs around information provision, service navigation, care coordination, and psychosocial support for dementia caregivers with cancer. The findings suggest that the experiences of dementia caregivers with cancer are not fully reflected in the existing cancer or dementia caregiving literature. By focusing on the intersection of these roles, our study provides new insight into the challenges and support needs of this overlooked group. Future research should evaluate interventions designed to support individuals managing both dementia caregiving responsibilities and a personal cancer diagnosis and treatment.

## Supplementary material

10.2196/93688 Multimedia Appendix 1 Interview topic guide for exploring the experiences of people facing cancer while caring for someone with dementia or memory problems.

## Abbreviations

COREQ: Consolidated Criteria for Reporting Qualitative
NHS: National Health Service
NIHR: National Institute for Health and Care Research
RTA: reflexive thematic analysis
