# Supplementary material for: Experiences of Informal Caregivers Facing Cancer While Caring for Someone With Dementia: Qualitative Interview Study
Source: JMIR Cancer. 2026 Oct 5;12:e93688. doi: 10.2196/93688 (PMC13637987; doi:10.2196/93688)
Supplement: Supplementary file 1 [file cancer-v12-e93688-s001.docx]

**Facing cancer whilst also caring for someone with dementia or memory problems.**

**Interview Topic Guide**

**INTRODUCTION**

A. Thanks

B. Any questions after seeing info sheet and consent form?

C. CONSENT - record on separate audio file

D. Socio-demographics and background: age, gender, ethnicity, working status, relationship to care-recipient.

E. Researcher provides an overview of the study and its aims.

1. Could you start by telling me about your family member or friend who you care for (or have cared for) who has/had memory problems or dementia?
   a. Type of dementia
   b. Length of time caring for
   c. Duties as a carer

**GETTING A CANCER DIAGNOSIS**

2. Could you tell me about your cancer diagnosis. How did you come to find out you had cancer?
   a. Symptoms or via routine screening.
3. Did juggling your role as a carer ever affect you being able to get a diagnosis? (e.g. go for tests)
   a. Issues/difficulties/challenges/complications faced with getting a diagnosis.
   b. Outcomes due to these (e.g. Did not attend/delays).
4. What helped you overcome/deal with that?
   a. Financial, emotional, practical, and informational support.
5. Who helped you overcome/deal with that?
   a. Family/friends/professionals (NHS/Social care etc).
6. How did that help /benefit you?
   a. Helped with caring for PLWD.
   b. Helped with getting a cancer diagnosis (e.g. rearranged apt slot).
7. What else could have been done to help you with getting a cancer diagnosis?
   a. Financial, emotional, practical, informational support.
   b. By whom: Family/friends/professionals (NHS/Social care etc).
   c. How could this be best done.

**HAVING CANCER TREATMENT**

8. Could you tell me about any cancer treatment(s) you have received?
   a. Type and treatment (i.e. surgery, chemo, radiotherapy etc.)
   b. Side effects – impact on caring for PLWD.
   c. If none – reasons for not having treatment (Go to Q12)
9. Did juggling your role as a carer ever affect you being able to have your cancer treatment?
   a. Issues/difficulties/challenges/complications faced with having treatment.
   b. Outcomes due to these (e.g. Did not attend/delays).
10. What helped you overcome/deal with that?
    a. Financial, emotional, practical, and informational support.
11. Who helped you overcome/deal with that?
    a. Family/friends/professionals (NHS/Social care etc).
12. How did that help benefit you?
    a. Helped with caring for PLWD.
    b. Helped with getting a cancer diagnosis (e.g. rearranged apt slot).
13. What else could have been done to help you with your cancer treatment?
    a. Financial, emotional, practical, informational support.
    b. Family/friends/professionals (NHS/Social care etc).
    c. How could this be best done.

**CLOSE**

14. On reflection now, what advice (top tips) would you give someone else who is facing cancer, whilst also caring for someone with memory problems or dementia?
15. Anything else they would like to say/discuss?

**Thank you for your time and for sharing your experiences and suggestions.**
